# Enhancing big data in the social sciences with crowdsourcing: Data augmentation practices, techniques, and opportunities

**DOI:** 10.1371/journal.pone.0233154

**Published:** 2020-06-10

**Authors:** Nathaniel D. Porter, Ashton M. Verdery, S. Michael Gaddis

**Affiliations:** 1 Virginia Polytechnic Institute and State University, Blacksburg, Virginia, United States of America; 2 The Pennsylvania State University, State College, Pennsylvania, United States of America; 3 University of California, Los Angeles, California, United States of America; King Abdulaziz University, SAUDI ARABIA

## Abstract

Proponents of big data claim it will fuel a social research revolution, but skeptics challenge its reliability and decontextualization. The largest subset of big data is not designed for social research. Data augmentation–systematic assessment of measurement against known quantities and expansion of extant data with new information–is an important tool to maximize such data's validity and research value. Using trained research assistants or specialized algorithms are common approaches to augmentation but may not scale to big data or appease skeptics. We consider a third alternative: data augmentation with online crowdsourcing. Three empirical cases illustrate strengths and limitations of crowdsourcing, using Amazon Mechanical Turk to verify automated coding, link online databases, and gather data on online resources. Using these, we develop best practice guidelines and a reporting template to enhance reproducibility. Carefully designed, correctly applied, and rigorously documented crowdsourcing help address concerns about big data's usefulness for social research.

## Introduction

Big data and computational approaches present a potential paradigm shift in the social sciences, particularly since they allow for measuring human behaviors that cannot be observed with survey research [1, 2, 3]. In fact, the transformative potential of big data for the social sciences has been compared to how “the invention of the telescope revolutionized the study of the heavens” [4]. However, some areas of social science have been slow to embrace big data. For instance, Lazer and Radford [5] note that only 15 of 422 articles (3.6%) published in the top journals in sociology between 2012 and 2016 contained analyses of big data. One reason why is “the need for advanced technical training to collect, store, manipulate, analyze, and validate massive quantities of semistructured data,” [6] training that remains nascent in many fields. But there are deeper, more fundamental constraints on the acceptance of big data among social scientists.

In this article, we make three points. First, we situate social science skepticism about big data in longstanding disciplinary concerns about validity and value. Though big data reveal many previously unseen elements of social life, they are often not created for research purposes, meaning that social researchers must assess whether measures derived from big data reflect their intended purpose (validity) and devise ways to incorporate big data into research questions of interest to social scientists (value). Second, we argue that the very features that make big data appealing as a novel source of information for social research–its size, granularity, and diversity–limit the application of traditional social science approaches to adding validity and value to orthodox sources of data, approaches which do not easily scale to the needs of big data in many research projects. Third, we consider a potential path forward: the use of online crowdsourcing techniques that blend traditional approaches to adding validity and value to social research and can be implemented at the scale necessary for use with big data.

Crowdsourcing is not the best solution to every data augmentation problem. Legal restrictions (such as the General Data Protection Regulation and Health Insurance Portability & Accountability Act) preclude certain crowdsourcing applications; such rules are complex and rapidly changing and outside the scope of this discussion. Both the treatment of workers and the content of the data itself may also raise ethical issues. While our discussion highlights certain ethical issues, as well as practical judgments of when crowdsourcing is likely to be useful, it is ultimately the responsibility of investigators to identify and address ethical concerns.

### Our argument: A roadmap

Despite its promise, big data’s perceived limitations cast uncertainty on its applicability in the social sciences. Many scientists have rapidly embraced big data because of the unprecedented information it makes available. Typical taxonomic efforts from computer scientists and others to delineate big data from traditional forms of data focus on these novel characteristics in what is called the “three Vs” framework [7, 8]: volume (or amount of data), velocity (or speed of data release), and variety (or data on rarely recorded activities). Volume, velocity, and variety are what make big data compelling and useful in a diverse array of fields.

All scientists are concerned with two other Vs: validity (or alternatively, veracity) and value [7, 9], but social scientists have been especially skeptical about the presence of these Vs in the context of big data. For social measurement, the presence of these additional Vs, which indicate authenticity or truth (validity) and what we can do with and learn from/of the data (value), is often difficult to assess and infrequently discussed in academic big data research [8, 9]. A search for “big data” in topics and titles indexed in Web of Science (2004–2019) reveals that most has come from research areas with foundational interest in the mechanics of data itself: Computer Science (61.4%), Engineering (37.7%), and Mathematics (13.5%). In these cases, social scientists may not be interested in the data itself, however, but the insight it may offer on social processes that produced it or result from it, and that task requires accessible means to assess its validity and enhance its value.

Characteristic of social science skepticism around big data are concerns that “the reliability, statistical validity and generalizability of new forms of data are not well understood. This means that the validity of research based on such data may be open to question” [10]. The type of big data we focus on does not come from a heavily theorized and well- planned scientific research project–they “are not the output of instruments designed to produce valid and reliable data amenable for scientific analysis”–which, at a minimum, creates discomfort among social scientists [5, 11]. Instead, it is a byproduct of other activity, which “has led some scholars to ask whether [big] data can provide anything beyond crude description” [12]. Without additional contextual information to help “tame” it, the concern is such data will remain too “wild” for answering valuable questions of interest in the academic social sciences.

Without clear approaches to quantify and increase the validity and value of big data, we believe social science skepticism will remain high. Researchers need to be convinced of the validity and value of big data, without adding substantially to its use cost, all of which we suggest can be accomplished through data augmentation. We define data augmentation as the process of (a) systematic assessment of measurement against known quantities or (b) expansion of existing data by adding new information. Data augmentation is a standard technique throughout the social sciences that can assume a manual or automated approach. Traditionally, these tasks are accomplished using trained research assistants (manual) or specialized algorithms (automated) to detect erroneously coded or poorly measured data (validity) or append existing data sources with new material (value). An example of a big data project that researchers manually augmented to increase validity is a study of posts made by high-schoolers on Twitter that mention bullying. The authors used two human coders to classify whether each post that mentioned bullying (or bullied, or bully, etc.) was a report of adolescent bullying or whether it represented some other use of the search terms [13]. Another example that used manual augmentation to add value is a recent study where researchers employed graduate students to code whether thousands of Tweets by U.S. Senators contained partisan messages [14]. On the automated side, Yin et al. [15] demonstrate how algorithmic approaches can increase validity. They propose a general means of separating human and automated (bot) accounts with high accuracy on Twitter based on a Bayesian detection model, which has the benefit of removing non-human actors from analyses. An example of automated data augmentation used to increase value is a well-known experiment on Facebook [16]. In this experiment, the authors examined how respondents’ purported emotions changed after being shown more purportedly positive or negative posts from friends. Emotions and their associated positivity or negativity were assessed by applying a sentiment analysis method to the words used in posts. Sentiment analysis, in this case, serves as an automated way to gain additional information about big data (the posts), augmenting its value for research purposes. Of course, there are many more examples of both manual and automated approaches to data augmentation to add either validity or value or both [17, 18].

Unfortunately, data augmentation can be challenging to implement at the scale required for big data projects while addressing social science skepticism around issues of validity and value. The manual data augmentation in the aforementioned study of bullying, for instance, was only feasible because researchers examined a manageable number of posts (N = 7,321).

Automated augmentation approaches, such as adding value through sentiment analysis, are also difficult to implement without advanced training and may themselves be of questionable validity. The Facebook experiment discussed above has been criticized by social scientists for the augmentation being of unknown and potentially low validity [19]. Of course, the validity of automated data augmentation approaches can be assessed and potentially improved through manual data augmentation, as is becoming more commonplace in big data projects through procedures such as supervised machine learning [20], but the size and complexity of most big data would require substantial time and expense for knowledgeable trained coders such as graduate assistants to check.

In this paper, we argue that online crowdsourcing platforms can complement both manual and automated approaches to data augmentation, increasing the validity and value of big data in the social sciences at a low cost to researchers. We show that such tools are underused for non- experimental designs in the social sciences and that workers on these platforms can efficiently and effectively perform many data augmentation tasks including verifying automated coding, finding errors in embedded metadata, and resolving missing data. In other words, we argue that online crowdsourcing applications offer a scalable blend of manual and automated approaches to data augmentation that can easily be harnessed to increase validity and value for big data applications to social science research questions. We build this case in five steps: (1) review the use and perceived limitations of big data in the social sciences, (2) describe the online crowdsourcing process and its documented strengths and limitations as a platform for academic research, (3) investigate current practices in academic use of the largest online crowdsourcing platform, (4) conduct three case studies implementing online crowdsourcing to enhance ongoing sociological research and test the utility of crowdsourcing across different circumstances, and (5) draw on the above, as well as experiments embedded within the case studies, to produce evidence-based recommendations on when and how to implement online crowdsourcing to augment big data for best results. Finally, in light of the inconsistent and frequently incomplete reporting of online crowdsourcing procedures, we provide a recommended reporting template for online crowdsourcing as an academic data augmentation platform. We believe that this paper offers a clear roadmap for social scientists to begin incorporating more big data into their research designs in ways that directly address issues of validity and value. We conclude by reflecting on the strengths and limits of online crowdsourcing approaches to data augmentation for these purposes.

### Big data skepticism in the social sciences

Myriad actors such as corporations, governments, scientists, and even sports teams have embraced big data [21, 22, 23] but adoption has been slow thus far in many social sciences [5]. The literature indicates that the primary reason social scientists are making relatively rare contributions to big data research is that these fields hold deep skepticism about data that is not designed for academic research [5]. Even those optimistic about the promise of big data critique its validity and value, including its lack of standardized reporting [24], poor measurement [25], decontextualization [20], and tendency toward “big data hubris” [11] that ignores threats to validity [26, 27]. Generalizability is another concern; most big data studies do not proceed with a clearly conceptualized population to which inference can be made [5, 28, 29, 30]. Disciplinary divisions in computational skills [31, 32, 33] and epistemology pose additional challenges [34], as do divides between industry and academic research [28, 35]. However, federal funders and several universities have funded a wide range of new training programs and other undertakings at the nexus of big data and the social sciences that may, over time, alleviate these pressures.

The broad range of concerns about big data from social scientists has led to a number of reflections on what steps can be taken to address this skepticism. However, our reading of the literature indicates that these reflections have focused more on the issues of generalizability than other, equally important concerns. For instance, in their review article, Lazer and Radford [5] list the vulnerabilities of big data research in sociology. The primary listing–indeed the “core issue”–is generalizability, “… *who* and *what* get represented” [5]. While these authors do acknowledge validity and value concerns, they are given only marginal discussion. Among the smaller number of studies paying careful attention to validity and value, there is a belief that they constitute a minority. Tufekci [36] details specific concerns about the validity and value of many social media analyses, also broadly true of other big data applications. These include platform bias, selection on the dependent variable, "algorithmic invisibility" (511), and intangible "field effects" (505). We argue that the oversight Tufekci observes is symptomatic of a fundamental gap between what researchers worry about with big data and what is being done to address those worries.

In general, the primary means of assessing and increasing the validity and value of data in the social sciences is undertaken through data augmentation. Examples of past big data augmentation include converting less structured data to more analytically tractable forms, linking multiple existing data sources [37] or collecting additional variables to check for spurious relationships or causal mechanisms [12, 38]. As reviewed above, there are both manual and automated approaches to data augmentation, but neither is likely to be sufficient to both scale to the problems posed by big data and address social science skepticism about it. Instead, we focus on a third option that can enhance both automated and manual approaches to data augmentation: using online crowdsourcing marketplaces such as Amazon Mechanical Turk (MTurk). Our work thus seeks to popularize and formalize a new tool within the nascent set of methodologies designed to increase the value and validity of online data collection efforts [12, 39].

Online crowdsourcing is less technically demanding than automated approaches and can provide supplemental evidence of accuracy based on user judgment or augmented comparison with outside sources or both. Compared to common manual approaches, MTurk is nimbler and less costly, allowing increased scale of augmented analysis. Compared to purely automated approaches or even blended approaches like supervised machine learning, online crowdsourcing through MTurk has the ability to produce well-understood measures of validity like inter-rater reliability or to merge data with sources that are not amenable to automated discovery, as well as retaining the reassuring feature that actual human beings have examined the coding. While some social scientists are using MTurk for research [40, 41, 42], we argue that formalizing this approach to data augmentation will expedite the widespread acceptance of big data in the social sciences and overcome barriers to its application. In the next section, we review MTurk as a promising research platform that we argue allows researchers to undertake big data augmentation at scale more simply, quickly, and cheaply than data augmentation through traditional automated or manual approaches.

### MTurk as a research platform

The name “Mechanical Turk” is derived from the 18th century chess-playing “machine” commonly known simply as “the Turk”. The Turk consisted of a complex cabinet of gears with a magnetic chessboard on top and a model of a human similar to a mannequin dressed in Turkish robes with a turban. Human chess players could play against the “machine” and would often lose. The Turk toured Europe and the United States throughout the late 18th and early 19th centuries. However, the Turk was a hoax as it was not an automated machine but rather an elaborate fake with a man inside playing the actual chess game [43, 44]. Amazon named their own version after the original Mechanical Turk to indicate that humans can still do things that computers cannot. Amazon’s MTurk is an online crowdsourcing marketplace that brokers what MTurk parlance refers to as Human Intelligence Tasks (HITs) between requesters and workers. The idea of a HIT is described succinctly by Amazon:

Amazon Mechanical Turk is based on the idea that there are still many things that human beings can do much more effectively than computers, such as identifying objects in a photo or video, performing data de-duplication, transcribing audio recordings, or researching data details. Traditionally, tasks like this have been accomplished by hiring a large temporary workforce (which is time consuming, expensive, and difficult to scale) or have gone undone.

Anyone eligible for employment in the U.S. or India can work on MTurk, although task completion requires reliable internet access. U.S.-based MTurk workers are on average younger, more educated, wealthier, more technologically savvy, and less racially diverse than average Americans [45, 46, 47]. As such, many worry that samples drawn from MTurk are less representative than population based surveys [45], though not as fraught as convenience samples [48].

However, when considering MTurk as a big data augmentation platform, rather than a population to sample and survey, we argue that work quality matters more than worker representativeness. MTurk workers tend to pass screening tests at high rates [45] with high reliability between [49] and within workers [50]. At the same time, recruiting workers for data augmentation tasks through MTurk has three major limitations. First, workers lack specialized area knowledge; second, they cannot access restricted information (e.g. workers cannot download most academic journal articles); and third, MTurk compensation is based on task completion, not time, which presents challenges for fielding complex, judgment-based tasks [46, 51]. We return to these ideas below. For now, it is worth noting that these limitations mean that crowdsourced tasks are most appropriate for data augmentation when they can be broken into *concise* and *unambiguous* chunks using *open-access* information.

### MTurk in the academy: A content analysis

MTurk is popular with academic researchers; a recent report found that academics posted the plurality (36%) of all HIT groups during the study period [52]. Academics have hailed MTurk’s low costs and rapid results, and even expressed cautious optimism about it as a survey platform [30, 53]. Its feasibility and reliability for data augmentation, however, remains unexplored.

To better understand how academics use MTurk, especially for data augmentation, as well as how they report on such use, we conducted a content analysis of a random with-replacement sample of 150 articles from Web of Science matching the topic search “mechanical turk” and published between 2011 and 2018. The search returned 1,684 total records that we then sampled. We removed 19 matches, 11 that did not use MTurk and 8 where full-text access was not available, yielding a final sample size of 129 articles (124 unique; statistics below are weighted for replacement sampling). In the online supplement, we provide metadata about these articles. We address three questions in this content analysis: a) who uses MTurk for academic purposes, b) what is it used for, and c) what details are reported about the use of the platform.

Table 1 reports fields where articles in our sample were published. A plurality (41%) of the papers we examined were in psychology and related fields (psychiatry and social psychology), followed by allied health sciences (16%), with five other fields comprising at least 5% of the sample. Table 1 shows proportions and counts for all fields.

**Table 1:** Sampled articles using MTurk by field, 2011–2018.

Field	Example Disciplines	Proportion of Articles in Sample (Count)
Allied Health Sciences	Medicine, Biostatistics, Public Health, Nutrition	0.155 (20)
Business and Economics	Business, Finance, Accounting, Economics	0.062 (8)
Communication and Culture	Communication, Cultural Studies, Linguistics	0.039 (5)
Engineering and Computing	Engineering, Computer Science, Statistics, Educational Technology, Information Systems	0.054 (7)
Law	Law, Legal Studies	0.031 (4)
Leadership and Organizations	Management, Leadership, Organizational Science	0.062 (8)
Marketing Sciences	Marketing, Tourism, Hospitality, Fashion	0.062 (8)
Neuroscience	Cognitive Science, Neuroscience	0.016 (2)
Politics, Administration and Policy	Political Science, Conflict Resolution, Policy, Public Administration	0.078 (10)
Psychological Science	Psychology, Psychiatry, Social Psychology	0.411 (53)
Sociology and Anthropology	Sociology, Gender Studies, Anthropology	0.031 (4)

Article counts grew steadily from MTurk’s founding in 2011 through 2016 and have remained high; 70% were published between 2016 and 2018. In general, these articles are cited frequently, with Web of Science’s citation counts indicating a mean of 19 citations (11 after removing one article with over 500 citations) for articles at least two years post-publication. These levels compare favorably to general article citation counts across many fields, where citation counts often average one per year or less.

We are also interested in what researchers use MTurk for, specifically how often it is used for data augmentation. Table 2 reports on the types of tasks academic researchers assign to MTurk workers. Because of psychology’s disproportionate use of MTurk, we disaggregate results by whether the article was in a psychological field. Most papers used MTurk to conduct surveys (66%), frequently with an embedded experiment (43%), although non-experimental surveys were more common in psychology (40%) than other disciplines (12%). In our sample, data augmentation was much rarer for both psychological (23%) and non-psychological (16%) studies. Of the studies involving data augmentation, workers are most often asked to perform tasks replicating other data, such as lab experiments (16%). Less frequently, they are asked to code data provided by the investigator (6%) or elaborate it with additional information (9%). In none of the studies were workers asked to collect publicly available data from the web.

**Table 2:** Worker tasks in articles using MTurk by field, 2011–2018.

	Psychology (n = 53)	Other fields (n = 56)	Total (n = 129)
*Take Surveys*	79%	57%	66%
Estimate population or subpopulation values	6%	5%	5%
Pilot survey for items or scales	11%	10%	10%
*Experimental Designs*	47%	63%	57%
Survey experiment	40%	45%	43%
Cooperative or interactive experiment	1%	20%	15%
*Data Augmentation*	23%	16%	19%
Verify/Replicate Other Data	17%	16%	16%
Elaborate on Data Provided by Researcher	6%	12%	9%
Code Factual Data Provided by Researcher	0%	10%	6%
Collect Publicly Available Data from Web	0%	0%	0%

Many studies ask workers to complete multiple tasks, so major categories percentages do not add to 100%.

Another question of interest is how academic researchers report on their use of MTurk to improve transparency and replicability, ensure quality of data augmentation or other tasks, and verify that workers are treated ethically. We found gaps in reporting standards that may impair the validity, value and replicability of MTurk as a data augmentation tool. Nearly every article we examined (98%) described data collection procedures like HIT content in detail, and most (86%) included at least basic summaries of worker demographics. However, few articles we examined reported required worker qualifications, criteria for work rejection, or validation criteria. Only 8% met what we define as minimal reporting standards across all three key areas for peer evaluation and replicability: a) a detailed description of the HITs and process (36%), b) information on worker qualifications, acceptance criteria and pay (25%), and c) descriptive statistics or multivariate analysis to evaluate sample characteristics (64%). We include more details on these standards below in the best practices section, our suggested reporting template, and in the online supplement.

The results of our content analysis highlight that academic use of MTurk is largely limited to experimental studies and surveys. In contrast to this typical use, we advocate that researchers expand their use of MTurk for data augmentation, which will have particular benefits for social science applications of big data that wish to address concerns about validity and value. We found that researchers are beginning to do this, but they do not offer enough detail on the process for formal evaluation or replication. In light of these opportunities and challenges, the remainder of this article examines three case studies and focuses on developing clear, evidence-based best practice guidelines on when and how researchers can successfully augment data with MTurk and report on doing so.

## Case studies

We now present three case studies that apply MTurk to diverse sociological subfields to augment big data (cases 1 and 2) or test MTurk’s data augmentation capacities against known benchmarks from ongoing sociological data collection (case 3). These cases allow us to compare MTurk to other data augmentation approaches, both automated and manual. For cases 1 and 3, we collected analogous data automatically and manually, enabling validity comparisons. We also embedded design experiments in cases 2 and 3 to test how HIT design and implementation can affect cost, quality, and worker experience. Our goal is to develop insight for the benefits of big data augmentation through online crowdsourcing and how researchers can best move forward with such projects. The data from these case studies are not optimized for external validity in the sense of reuse in other contexts, but rather are selected as real-world applications of data augmentation in MTurk to avoid the need for expert coding of large samples. Our goal here is to demonstrate crowdsourcing as a tool for rapid use-specific data augmentation.

We designed all HITs based on past recommendations [45, 47, 48] and revised according to common worker concerns voiced in the popular MTurk forum turkernation and our own pilot studies. We collected all data between October 2015 and July 2016. The online supplement provides full versions of instruments and de-identified results.

### Study 1: Academic affiliation—Overview and methods

Our first case shows how MTurk can enhance the validity of big data. It is part of a larger project on the role of interdisciplinary dissertation committees in knowledge production [54]. The original project used an algorithm to code the academic field of faculty based on their roles in doctoral committees. For instance, if a faculty member chaired committees in one field and was a member of committees in another, the algorithm assigned them to the field in which they chaired. Most cases were less clear cut, however, and required more complex assignment rules reviewed in greater depth in the original paper. Such algorithmic assignment indicated a surprising proportion (56%) of interdisciplinary dissertation committees. The credence given to these prevalence statistics, however, hinges on the accuracy of the automated coding. This represents a classic concern voiced by social science skeptics about automated augmentation of big data. For instance, compare the critique of sentiment analysis in the aforementioned Facebook experiment [16, 19] or concerns about search term inclusion in Google Flu [11, 55]. Manually verifying a sample–manual data augmentation–represents one way to check result validity, however, our tests indicated that finding and hand coding the fields of a sample of 2,000 of the 66,901 faculty (3%) would have demanded over 230 hours of trained coder work. This time commitment translates to more than three quarters of a semester of typical graduate research assistant support, assuming a 15-week semester at 20 hours a week.

Rather than training graduate student or other internal coders to verify these results, we tested the data augmentation capabilities of MTurk. We did so by creating three sequential tasks that split the process of validating the algorithmic coding of faculty members’ fields into discrete steps. First, we asked workers to find the departmental webpages of a random sample of faculty members using a search link that limited results to the official website of their academic institution (see Discussion and S1 Appendix for details). This step provided a sample of faculty whose academic field could be externally validated. Second, we asked workers to verify links obtained in task 1 and indicate whether each faculty member was listed in any of the 10 most common department names in the algorithmically coded field. This step helped to ensure that the links for specific faculty were correct. Finally, in the third task, we asked workers to evaluate whether any field on the faculty member’s page is associated with the field that was algorithmically assigned. For instance, if a faculty member listed “speech pathology” as their field and the assigned field is “speech and hearing sciences,” we aspire for workers to select that these fields are associated. This step constituted our primary interest, quantifying the validity of the algorithmic coding. We adapted all tasks from MTurk templates using the HTML and JavaScript programming languages, and collected them from separate but potentially overlapping pools of workers within the MTurk interface. A graduate research assistant invested approximately 40 hours in learning and managing this MTurk data collection. In all, we used MTurk data augmentation to check 2,043 automated classifications of faculty member fields, at a total cost of $590 including fees and pilot costs. Attaining comparable labor costs with a single graduate coder and no external validation would require pay less than $3.11 per hour, including any benefits.

### Study 1: Academic affiliation—Results and discussion

Were MTurk workers, operating without substantial oversight or prior training, able to validate the results assigned by algorithm? This case speaks to MTurk’s ability to add validity to big data, used here to confirm the automated coding of a large data set and bound rates of coding error. Table 3 summarizes the combined results for Case 1. Workers in the initial HIT successfully located 85% of faculty, mostly on preferred page types (faculty homepage, administrative list, or curriculum vitae). Subsequent workers flagged only 3% of URLs that prior workers submitted as referring to the incorrect person or institution. Of cases with unflagged URLs, workers identified 94% of faculty members as matching either the field or department we provided, which suggests that the original automated coding of these big data succeeded at a high rate, even allowing for the possibility of substantial worker error. Mean hourly worker pay in this case ranged from $7 to $16 and was higher for workers completing multiple HITs.

**Table 3:** Contingency table of HIT results for Study 1.

URL Found		Field Matched		Department Matched	
No	15.5%		NA		NA
Yes	84.5%	Unclear	3.9%	Bad URL	5.9%
				No	27.9%
				Yes	66.2%
		Bad URL	2.0%	Bad URL	34.3%
				No	22.9%
				Yes	42.9%
		No	13.1%	Bad URL	2.2%
				No	44.5%
				Yes	53.3%
		Yes	80.8%	Bad URL	0.9%
				No	12.4%
				Yes	86.7%

This case revealed some important lessons. Early pilots combined all stages (page location, department classification, and field classification) into a single HIT, but we found that workers took longer and gave flagged results more often in such conditions. With later pilots, we found that dividing tasks into the three steps outlined above minimized worker time and let us build in cross-verification tests where subsequent workers verified both the faculty web pages and affiliations provided by earlier workers. The conclusions of the original study hinged on the accuracy of machine-coded disciplines and fields. Using MTurk, we were able to empirically evaluate that accuracy with speed and cost-efficiency that could not be replicated with trained coders.

### Study 2: Linking to OpenLibrary—Overview and methods

**Fig 1:**
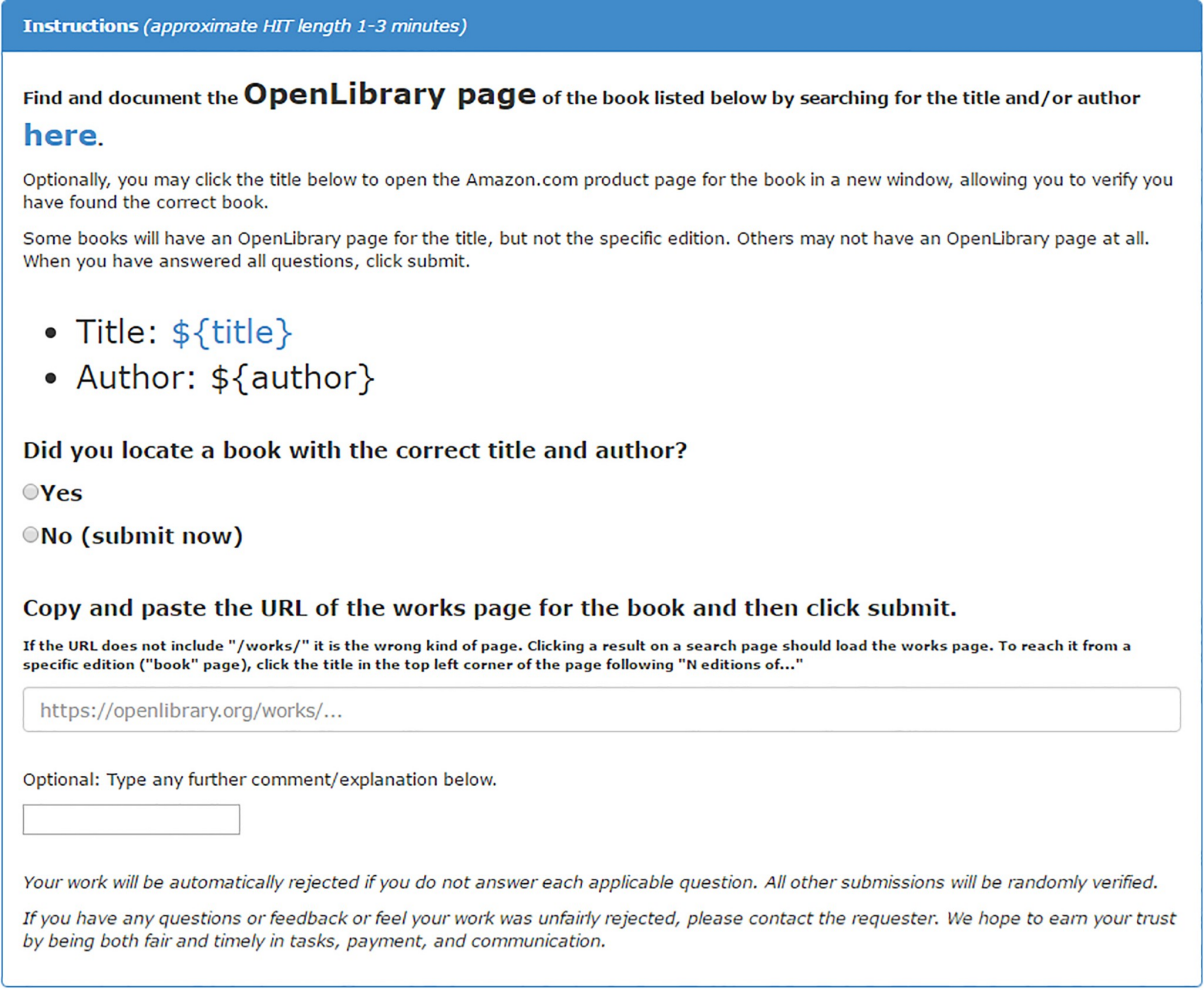
Experimental variant 1 for study 2 (complete).

**Fig 2:**
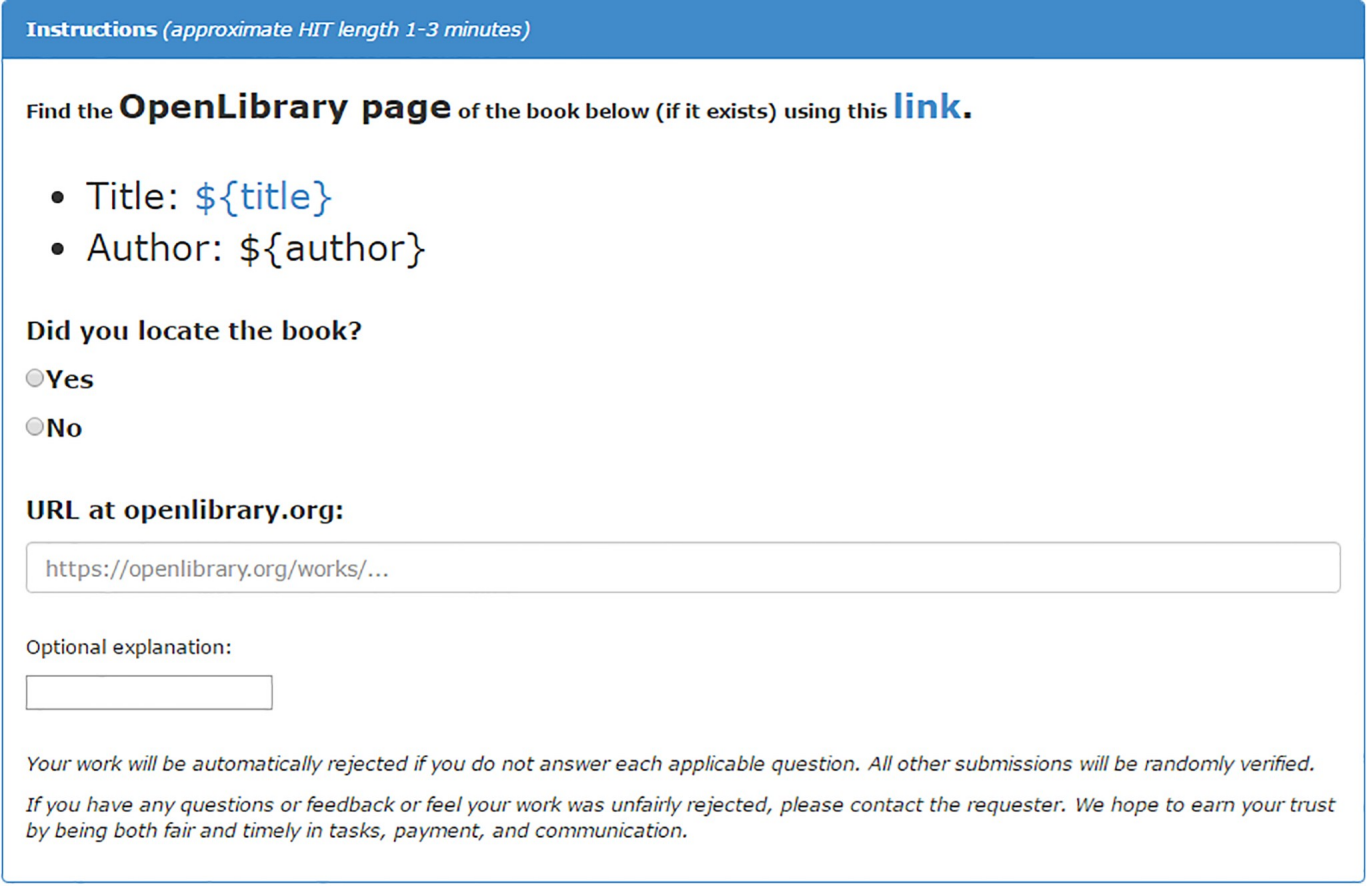
Experimental variant 2 for study 2 (brief instructions).

**Fig 3:**
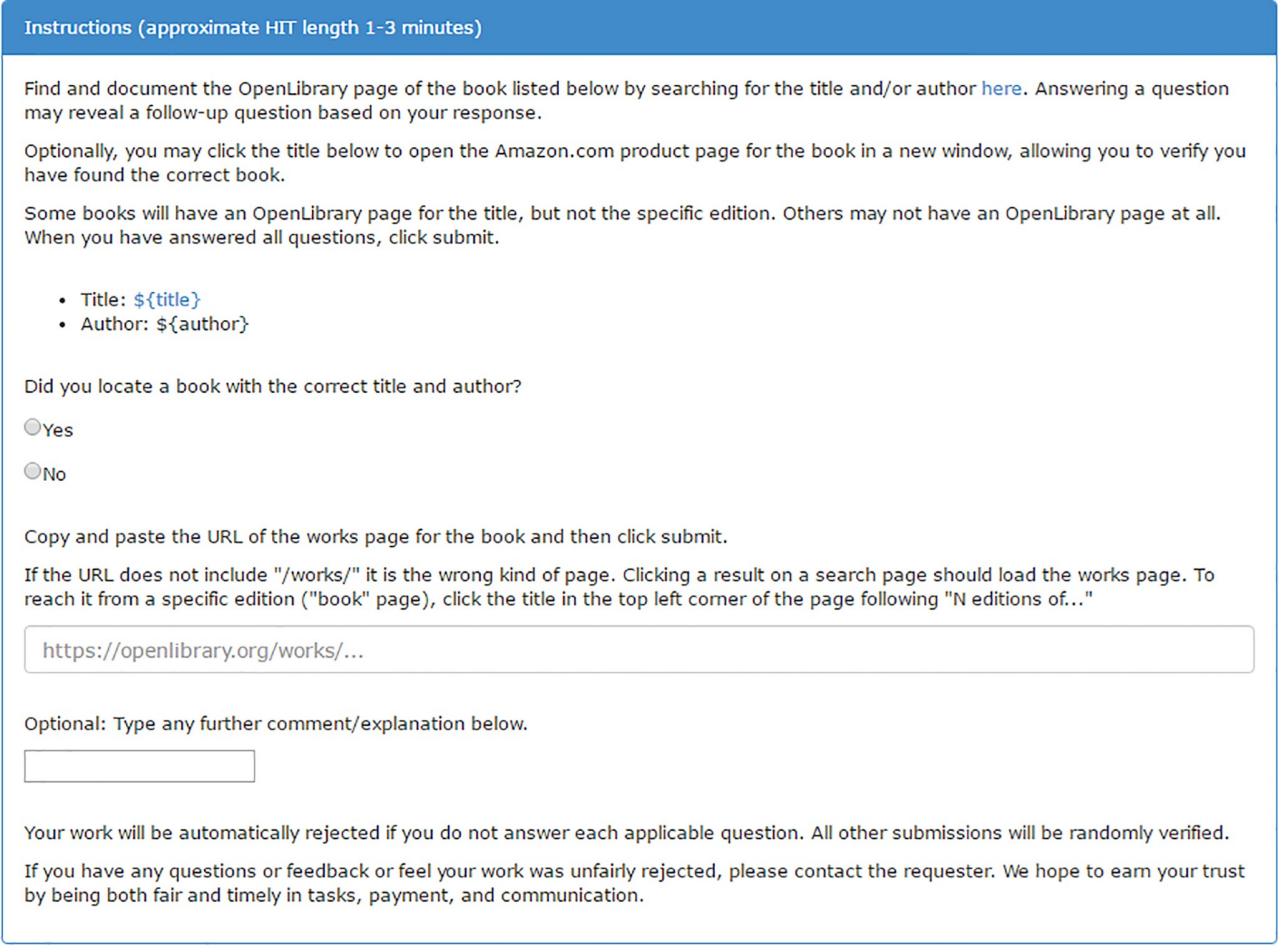
Experimental variant 3 for study 2 (plain design).

Our second case highlights how data augmentation with MTurk can enhance the value of big data. Here, we asked workers to link related data sources, and we experimentally tested how HIT design may affect work quality. This case builds on a project investigating book co- purchasing patterns connecting cultural groups, operationalized with retailer metadata scraped from the web. Unfortunately, necessary metadata were often incomplete, missing, or of questionable quality. For example, a book written by the founder of one Protestant denomination (Martin Luther) was listed as the top-selling item associated with a completely different denomination. To supplement missing information, we matched 1,055 (58%) books to additional metadata provided by OpenLibrary.org using international standard book numbers (ISBNs), a unique code identifying books. For 765 remaining unmatched books, we tested MTurk’s data augmentation capacities by asking workers to search for the books on OpenLibrary. As an experiment to determine means of improving HIT design, we randomly assigned each worker into one of three task variants. The first variant included full instructions with design features to enhance clarity (e.g. highlighting key text); the second used brief instructions but retained design features; while the third included full instructions with minimal formatting. Figs 1–3 provide screen shots of each condition; note that Amazon uses the ${variable name} notation as code to substitute values from input data provided by the requester (code available in supplemental files).

### Study 2: Linking to OpenLibrary—Results and discussion

Case 2 workers successfully found 283 potential matches (37%) for missing books in the original data. We followed up on HITs with comments and rejected submitted URLs outside the specified page types. A researcher checked every 20th HIT returned for accuracy during data collection and found very low rates of false matches (<1%) and false negatives (5%-10%). Checking during data collection (rather than using a simple random sample of all returned HITs) provides opportunity to save money by cancelling remaining unclaimed HITs if design flaws are discovered. Consistent with case 1, the 33 workers who completed only one task in this case averaged 298 seconds, but the 50 workers who completed multiple tasks averaged only 126 seconds per task. Total cost for this case including fees was $235.

The experiment embedded in this case illuminates how HIT design affects cost and quality. Workers presented with detailed instructions and design features spent less time per completed HIT (mean 171 seconds, S.D. 145) than those provided concise (230, S.D. 317) or minimally formatted (245, S.D. 233) instructions. Because of the small cell sizes in this task, such differences are not significant with two-tailed T-tests; nonetheless, we take the magnitude of the differences to indicate that better instructions are likely to yield better results. Though there is a general concern that paying workers per task may lead them to rush and skim longer instructions, yielding lower quality work, we did not find that this approach compromised accuracy in our testing. Instead, work accuracy in all three groups was high and statistically indistinguishable. We speculate that fuller instructions may reduce cognitive demands on workers and thus lead to lower completion times with comparable accuracy. By connecting retailer data to third-party data on the books in the study, MTurk provided means not only of verifying the validity of retailer topic coding, but also augmenting analysis with topic-modeling and additional layers of networked relationships between books using OpenLibrary data.

### Study 3: Mental health websites—Overview and methods

Our third case study does not focus on a big data project directly. Instead, it tests the possible extent of MTurk’s data augmentation capacities and directly evaluates MTurk data augmentation against a “gold standard” benchmark from a set of trained coders in an existing sociological data set. This case reveals how task complexity affects MTurk results and it provides alternate methods of assessing the quality of MTurk data augmentation. In this case, we compare the performance of trained coders against MTurk workers in a study of college student mental health. The Healthy Minds Study Institutional Website Supplement (HMS-IWS) collects data on 74 topics across 8 areas related to resources, information, and the presentation of information on mental health services from college and university websites. It is, itself, adding value to a standard survey (the Healthy Minds Study) [56, 57, 58] through manual data augmentation.

For three years, the HMS-IWS team, including a Ph.D. researcher and two trained graduate research assistants, each coded relevant items from institutional websites. There is high inter-rater reliability in this manual data augmentation approach but also extensive costs and time. In this case study, we asked 40 MTurk workers to record information from one of three college or university websites. We provided workers with a brief explanation for each task (see S1 Appendix) as well as the website link. We varied HIT construction across four categories to test how HIT organization and design affects work quality and cost. In HITs 1A and 1B, we gave workers a set of 21 items (18 yes/no and 3 open-ended) spanning four broad categories (general information, campus-specific information, information for individuals other than students, and diagnosis) and paid $1.50 for the task. In HITs 2A and 2B, we gave workers a set of 33 items that fit under a single category (services and treatment), including 30 yes/no and three open- ended questions, and paid $1.75 for the task. Finally, we varied the HITs between versions A and B, with the sole difference between versions being the addition of a paragraph in the B variants that told workers we would check accuracy and that users with too many inaccurate answers would not receive payment.

### Study 3: Mental health websites—Results and discussion

To evaluate worker accuracy, we compare results from MTurk workers to results from the trained coders, which we take as a gold standard benchmark for accuracy. Three trained researchers first coded each of the 48 binary items for each of the three websites. The researchers initially agreed on 131 of the 144 total items (90%) across the three websites, and the remaining 13 items were rechecked until consensus was reached. In contrast, MTurk workers correctly answered binary items at a rate of 63% for HIT 1A, 70% for HIT 1B, 78% for HIT 2A, and 82% for HIT 2B. Given the binary response choices, these rates are generally low. Consistent with longstanding findings in statistics [59, 60], using a majority vote decision rule to aggregate MTurk responses to the same question correct would have resulted in errors for 31% of items. The accuracy difference between HIT 1A and HIT 1B is significant using an unpaired t-test (p<0.05), while the difference between HIT 2A and HIT 2B is not significant under the same test. The pooled difference between HITs 1 and HITs 2 is also statistically significant (p<0.001). Moreover, the pooled results show that individuals given the A variants were more likely to have a low accuracy rate than those seeing the B variants at a rate of 22% to 8%, respectively (p<0.05).

In evaluating this case, we discovered an additional finding that pertains to best practices for MTurk data augmentation. Researchers might be tempted to proxy data quality with task completion time, discarding work completed in the shortest or longest amount of time, or both. However, we found little benefit from doing so. The correlation between accuracy and completion time is 0.34, and falls slightly (to 0.29) if we remove work completed in the bottom decile of completion times. If we remove work completed in the top decile, it increases (to 0.48). Removing both changes the correlation only marginally (to 0.44). On this basis, we conclude that completion time is a weak indicator of work quality. Some who complete the task quickly may simply be good at it, while some taking the longest amounts of time may have stepped away from the computer or worked on multiple tasks at once without sacrificing work quality. Recall that MTurk workers are paid by the task, not by completion time.

Overall, results from this case show that not all data augmentation tasks can be done effectively by online crowdsourcing workers. We focused on simple yes/no questions and received a 63% accuracy rate in one HIT iteration, only marginally better than random chance.

However, we can draw other important conclusions about using MTurk for data augmentation from this case: alerting workers to the possibility of payment loss from sloppy work improves accuracy [61], as does the careful ordering of work into logical groups. Finally, researchers should be careful when evaluating work accuracy, as high error rates were maintained under consensus coding and showed little relationship to completion time.

## Discussion

The use of online crowdsourcing for survey and quasi-experimental research is gaining acceptance in the social sciences. A series of studies that compare the results of parallel surveys and experiments using MTurk and traditional methods have evaluated online crowdsourcing with generally positive assessments [29, 30, 45]. Our content analysis of published social science papers that use MTurk indicated that such evaluations have generated a set of informal norms around design and reporting for quasi-experimental and survey-style MTurk studies.

We argued that online crowdsourcing as a data augmentation platform holds unique potential to add validity and value to applications of big data to social science research questions at low cost, and our content analysis suggests that researchers are beginning to use it for these purposes. However, in contrast to the emergence of norms for experimental and survey research with online crowdsourcing platforms, we found little evidence of standards for the design and reporting of data augmentation with such tools. We addressed that gap in the literature by presenting a series of three case studies designed to consider specific big data augmentation challenges, test MTurk data augmentation against known benchmarks, and improve the research community’s understanding of best practices of data augmentation through online crowdsourcing.

In this section, we consider the implications of both the content analysis and our three case studies in the context of past recommendations about online crowdsourcing for academic research. We aim to provide evidence-based guidance for researchers in two situations: (1) those exploring the viability of online crowdsourced data augmentation for a project, and (2) those seeking to improve the validity and value of data augmentation efforts with online crowdsourcing. While we believe this guidance will be most useful to researchers seeking to apply big data to social science research questions, we think that they may be of interest to researchers conducting more traditional social science analyses as well. Finally, we hope that future researchers, reviewers, and editors will find these considerations useful when evaluating data quality, reporting adequacy, and replicability in online crowdsourcing studies. To advance that goal we offer a model reporting template in the S1 Appendix.

### Strengths and limitations of using online crowdsourcing for data augmentation

Our three case studies test whether and when online crowdsourcing is practical for adding validity and value to big data projects. We found that data augmentation through online crowdsourcing platforms performs best in instances like case 1, where target data are clearly defined and standardized, but it is too time-consuming, challenging, or costly to automate information discovery or for trained coders to manually recover and evaluate this information. In such tasks, workers on online crowdsourcing platforms can find and code information quickly and efficiently. The results of case 2 suggest that researchers must consider the importance of the specific output data and likely return on investment before fielding HITs. While results in this case were accurate, most books lacked a match, reducing the effective value of data augmentation through online crowdsourcing. Nonetheless, were this case focused on a larger project with tens of thousands of missing records, for instance, gains could be substantial. Case 3 looked at MTurk’s potential for research beyond simple data augmentation tasks, but it offers a more cautionary tale, wherein the non-specialized skills and task completion incentives of online crowdsourcing workers led to poor accuracy. While data augmentation through online crowdsourcing may not satisfy the complex needs of standard sociological studies such as the HMS-IWS, it can still save time and cost when used for smaller, more straightforward portions of the data collection process that would be necessary with data augmentation.

To the extent that each of the following are true, we argue that using online crowdsourcing for data augmentation should be considered more beneficial for potential cost and time savings:

1. Data collection cannot readily be automated.
2. Data can be found and/or coded by web-savvy persons without special training or knowledge.
3. Analytic needs for data are factual and do not include population estimates or comparisons with under-represented groups (minorities, individuals outside the US/India, older Americans, etc.).
4. Factual tasks can be split into smaller chunks without substantial duplication of effort.
5. Rapid results and the ability to test alternative instruments (e.g. pilot tests) are advantageous.

### Best practices for academic requesters

Given the broad range of goals, methods, and tools used by academic requesters, this section provides evidence-based guidance for maximizing the validity and value of data augmentation using online crowdsourcing marketplaces. It assumes a researcher’s goal is data augmentation, but it is also broadly applicable to surveys and experiments, with differences as noted. Once the decision has been made to use online crowdsourcing for data augmentation, a typical workflow includes three phases: design, collection, and analysis.

The design phase is most critical; it sets conditions for success in subsequent phases. Clear visual design and precise, jargon-free instructions increase worker efficiency and lower the post-collection burden on requesters to manually check data quality. Based on experimental tests in cases 2 and 3, we recommend providing comprehensive instructions and examples, but highlighting (through size, color, placement, etc.) the most important instructions for task success, as well as how work will be evaluated in payment decisions. Formative pilot studies can help to identify problems with design. If using external tools, such as pairing MTurk with survey administration platforms, it is vital to pretest HITs and ensure the correct operation of validation processes for external task completion. Malfunctioning codes are a common complaint on worker forums, as workers who have invested as much as an hour in a survey may be unable to receive compensation. We recommend pre-testing all HITs on the requester sandbox (http://requestersandbox.mturk.com) and testing codes as part of this process.

Clear design for search or evaluation tasks faces the additional challenge of user customization and personalization. Major internet search engines often customize results based on user location and past search history. Requesters seeking to collect data that are comparable across cases should minimize variability by embedding custom search links in the directions, using non-personalized search engines such as DuckDuckGo, as we did in case study 1, and specifying how many results to use (e.g. the first 20). Search links can contain elements from the input that vary between cases, embed Boolean logic, and restrict results to specific domains.

Cases 1 and 3 demonstrated two additional principles specific to data augmentation and other factual HITs: a) iterative data collection, and b) related task grouping. Iterative data collection favours rapid and efficient collection of a limited range of data over single-shot data collections designed to answer numerous questions. With large online crowdsourcing marketplaces, a sizable labor force is always available, and researchers can easily integrate prior task output into subsequent input. Outside of tasks requiring extensive setup or training, delaying follow-up questions to later tasks or collecting data for a sample rather than every case poses little threat to data quality. The ease of redeployment and incremental expansion generally make it better to wait when unclear whether a researcher will need a specific piece of information, preparing follow-ups as necessary.

We refer to the splitting of work into smaller and more coherent tasks as related task grouping and advocate that it improves work quality. Compared to initial single-shot versions of study 1, splitting the design into three HITs decreased cost and improved accuracy. Smart chunking lets workers self-select into tasks and not feel constrained to finish a longer task poorly to avoid sunk time. In both studies 1 and 2, a small proportion of the total number of workers completed most HITs, spending less time per HIT with at least equal accuracy. Related task grouping also avoids overpaying for work that is not completed. For example, a common application of big data augmentation through online crowdsourcing is asking workers to answer questions about a specific web link. If the link is invalid, any subsequent questions are inapplicable. If finding the initial links is also a goal, devoting a single task to identifying a suitable web address and asking subsequent workers to verify web address accuracy can save on excess pay while also providing cross-verification of the initial task’s success.

Big data augmentation with online crowdsourcing is often swift and hands-off once HITs are posted, but some simple steps before, during, and immediately following HITs can improve data quality and requester reputation. Before activating a HIT, requesters can freely specify minimum worker qualifications, such as by only requesting workers with evidence of past task success or who have completed pre-tests [62 and 63 discuss tools for requesters more extensively]. Requesters should also monitor their registered email during and immediately following HIT batches, as workers may contact them when they are unsure about the appropriate response, to report unclear directions or glitches, and to appeal rejections. Many circumstances, including browser malfunction, accidental user error, or common mistakes can result in rejection of ambiguous or good work, so researchers often accept all complete HITs and later remove poor quality data.

Of the phases of online crowdsourcing implementation, scholars have paid the least attention to analysis and reporting. The variety of big data, their relative lack of structure, and the priority of computer science and engineering over the social sciences in the field have contributed to inconsistent reporting. For data augmentation with online crowdsourcing tools to increase the validity and value of big data, transparency is imperative as to the procedure used to collect the data, how their integrity was verified, and relevant information on workers.

We provide a recommended reporting template in the S1 Appendix with both standard items that should be included in reporting all online crowdsourcing studies and items to use in reporting specifically for big data augmentation. We recommend researchers report on key study features, its purpose and implementation, and the exact criteria that they used to determine data quality, including at least one of several potential validity checks. Whenever possible, we suggest that both instruments and output data should be made available through public data repositories, such as the Open Science Framework (osf.io) and the Dataverse network (dataverse.org) or other publicly accessible sites, such as Github repositories (github.com). In either case, standard confidentiality practices should be observed in removing unique worker numbers and other potentially identifying information before publishing data, and researchers must adhere to relevant human subjects research guidelines when appropriate.

Worker compensation is a final issue that deserves discussion. Typical worker compensation among the few academic studies that report hourly pay on MTurk is $1–2 per hour, rates that prior work suggests produce reliable results [48]. These rates, however, are far below U.S. minimum wages and legal only because MTurk workers are self-employed contractors not subject to minimum wage laws. Buhrmester and colleagues [48] found that compensation was not the most commonly cited motivation for workers, but recent findings suggest many workers rely on MTurk as primary or supplemental income [52, 64, 65]. We worry that such low payment rates can damage the broader research community by hurting the reputation of academic researchers. A 2014 experiment [66] estimated that HITs from requesters with good reputations in the online review forum Turkopticon recruit workers at twice the rate of those with poor reputations [64, 67]. We encourage researchers who wish to estimate costs to collect a small pilot study and target average hourly compensation of at least the U.S. federal minimum wage (currently $7.25).

## Conclusion

This paper offers data augmentation through online crowdsourcing as a scalable and low- cost means to address common concerns regarding the validity and value of big data in the social sciences. Whereas prior work has focused on the generalizability and ethics of big data, issues of validity and value have received considerably less attention. At the same time, while many have used online crowdsourcing marketplaces such as MTurk for drawing samples, or for experimental studies, few researchers have used them for data augmentation. In this paper, we attempted to bridge these literatures. We reviewed existing practices in academic research using online crowdsourcing and considered three empirical cases where big data augmentation through crowdsourcing enhanced ongoing research or illustrated the limits of data augmentation with such tools. Based on these analyses, we provided general guidance and best practices for academic research that uses online crowdsourcing for data augmentation and a standardized reporting framework. Although we emphasized the use of online crowdsourcing for big data augmentation, many of our findings and recommendations may be of value to researchers considering online crowdsourced labor for other tasks like fielding surveys. There is substantial promise in using online crowdsourcing to free up research assistant time without the need for highly-skilled programmers, and this paper offers some first steps to formalize knowledge about the potential for using these tools to help answer social science research questions.

## Supporting information

S1 Appendix Reporting template how to use. (DOCX)
