# Supplementary material for: Enhancing big data in the social sciences with crowdsourcing: Data augmentation practices, techniques, and opportunities
Source: PLoS One. 2020 Jun 10;15(6):e0233154. doi: 10.1371/journal.pone.0233154 (PMC7286483; doi:10.1371/journal.pone.0233154)
Supplement: S1 Appendix — (DOCX) [file pone.0233154.s001.docx]

# Appendix 1: Reporting Template How to Use

This template provides a simple and standardized format for reporting Amazon Mechanical Turk (MTurk) results in the social sciences. We anticipate the template may be usable for other crowdsourcing platforms with only small modifications, but focus on MTurk as the largest and most established platform for academic use. This version is a minimal reporting template, including a recommended set of quantities to allow reviewers and readers to evaluate the general quality of the data, its applicability, and possible limitations or problems. Because of the variety of possible uses and structures of MTurk studies, investigators are encouraged to report additional details not anticipated in this template as necessary. Items in the first section should be included in all studies reporting MTurk results. Items in the second section should be included whenever germane to the design of the study. We encourage investigators to maintain a public repository with this documentation, copies of all instruments, and (when possible) an anonymized copy of the original output. Recommended locations for repositories are within online supplements to an article, open-access data archives, institutional repositories, or public GitHub repositories. The online supplement includes a sample of such a repository containing all recommended material for the case studies we review in this paper. It additionally includes (1) data and further information on the formal content analysis and (2) a suite of freely adaptable Stata scripts to help prepare raw MTurk output for analysis and public archival.

# Template for Reporting Social Scientific Data Collected using Amazon Mechanical Turk*

| **Recommended for all studies** | |
| --- | --- |
| **Item** | **Description** |
| Batch | Name or signifier of batch |
| HITs | Number of HITs (unique cases in input file) |
| Workers per HIT | Number of workers assigned to complete each HIT (e.g. provided identical input) |
| Date(s) | The date(s) and time period during which the batch was collected |
| Instrument(s)+ | HTML, complete description, or screen capture of instrument(s) for tasks exactly as implemented |
| Source of input data | What defines cases in the input file and where the data are originally derived from |
| Output variables | Descriptive statistics for output variables used in analysis (including missing patterns and worker demography if applicable) |
| Qualifications | List of requirements for workers to accept HITs (standard or custom) |
| Rejection criteria | Description of how decision was made to approve or reject assignments |
| Rejection rate | Proportion of submitted assignments that were rejected |
| Validation check(s) | At least one additional procedure (other than qualifications or rejection criteria) to verify data quality. Such procedures include:   - Consistency between multiple workers on the same HIT (inter-rater reliability) - Accurate completion of items with known correct answers included in HIT - Worker attention checks (questions with obvious correct answers to ensure workers are reading questions and following directions) - Confirmation in later sequential HITs - Consistency with another method (e.g. automated coding or trained coders) |
| **Recommended whenever applicable** | |
| **Item** | **Description** |
| Privacy protections | List any relevant privacy regulations or concerns and methods ensure legal compliance and ethical fairness |
| Third-party tools | Name and version number (or date, if non-versioned) of any third party tools such as Qualtrics or SurveyMonkey used to administer HITs externally |
| Design features | Precise description of any contingency, experimental, or quasi-experimental design that is not clear from the instrument (often requires third-party tool) |
| Sampling methodology | Information on any sampling process, including the population being sampled, how cases were selected for inclusion, and whether the sample is with replacement |
| Weights | List of any weight or adjustment variables and their derivation |
| Panel attrition | Standard panel attrition statistics for longitudinal data collection |
| Repeat worker rate | For surveys, experiments, and other tasks collecting information about workers, the proportion of HITs completed by workers who had already completed one or more HITs in the study |
| Repeat worker consistency | For tasks collecting information about workers, the proportion of demographic responses consistent between HITs by the same worker |

* Unless identical across batches, items should be reported for each batch of data collected using MTurk

+ We recommend these items be included in reporting table as the URL of an online repository
